# Carryover of neotame, WS-23, and other additives from popular disposable e-cigarettes to aerosol

**DOI:** 10.21203/rs.3.rs-11234503/v1

**Published:** 2026-10-05

**Authors:** Remi A. Mellinghoff, Isabel X. Maday, Elena M. Bouldin, Paul T. Anastas, Stephanie S. O'Malley, Suchitra Krishnan-Sarin, Julie B. Zimmerman, Hanno Erythropel

**Affiliations:** Yale University; Yale University; Yale University; Yale University; Yale School of Medicine; Yale School of Medicine; Yale University; Yale University

**Keywords:** Disposable e-cigarette, artificial sweetener, synthtic coolant, neotame, ENDS, WS-23

## Abstract

**Introduction:**

Disposable e-cigarette devices are popular among youth with fruit and candy flavored products dominating the market. While the addition of novel sweeteners and synthetic coolants has been quantified in the e-liquid of such devices, the relative amount of these compounds transferred into the aerosol and inhaled by users remains unknown. In this study, popular US disposable e-cigarette devices were analyzed for coolant, sweetener, and nicotine concentration, and subsequent carryover to aerosol was determined using an established protocol with cold-finger traps to collect aerosol.

**Methods:**

12 popular disposable e-cigarettes were identified and purchased between 2023–26, and 3 Sensa devices labeled as “0-nicotine” and 2 SpreeBar devices labeled to contain “Metatine” (6-methylnicotine) were also included to probe for the effect of nicotine on carryover. Menthol, nicotine, 6-methylnicotine (6MN), the synthetic coolants WS-23 and WS-3, and the artificial sweeteners neotame and sucralose were quantified in the liquids and the generated aerosol (20 puffs in triplicate) of 17 disposable e-cigarettes using GC-FID and LC-MS-MS.

**Results:**

Neotame and WS-23 were present in all 17 devices at concentrations ranging from 0.05–2.73 mg/g and 3.52–35.0 mg/g, respectively. Nicotine was found in 11/17 devices, menthol in 9/17, WS-3 in 4/17, sucralose in 2/17, and 6MN in 2/17. Mean carryover rates to aerosol were: Neotame 40 ± 13%, WS-23 84 ± 9%, nicotine 88 ± 12%, menthol 81 ± 8%, WS-3 88 ± 10%, and sucralose and 6MN carryover ranged from 26–54% and 72–77%, respectively.

**Conclusions:**

Although carryover rates to aerosol were lower for sweeteners compared to nicotine and coolants, users of these devices can be expected to be exposed to substantial amounts of the quantified 6 compounds. It is unclear whether neotame is thermally stable or breaks down during the aerosolization process. This study aims to provide a foundation for future toxicological and pharmacological studies that are needed to better understand the implications of chronic inhalation of neotame, WS-23 and the other tested compounds.

## INTRODUCTION

The market for electronic cigarettes in the United States has significantly shifted over the last decade. It has evolved from 1st generation “cig-a-like” devices and slightly larger “vape pens”, to bulkier “box mod” devices that were able to deliver higher amounts of nicotine to users. From there, “pod-based” devices were developed, which used a pH modifier to create so-called nicotine salts, allowing for a return to smaller and less powerful devices while still delivering high amounts of nicotine to users.^1,2^ The latest shift, which has occurred in the United States since February 2020, has been from pod-based devices mostly available only in tobacco and menthol flavors toward “disposable” devices that are being marketed in a wide variety of flavors.^3–5^ While no disposable e-cigarette has obtained marketing approval from the U.S. Food and Drug Administration (FDA) as of 8/2026,^6^ sales data suggests that disposable devices accounted for > 60% of the US electronic cigarette market in 2025.^7^

E-liquids, the fluids heated inside e-cigarettes to create the aerosol inhaled by users, are a complex mixture of chemicals including a carrier fluid (propylene glycol, PG; and glycerol, G), nicotine, flavorants (which includes coolants and sweeteners), and other additives such as pH modifiers.^8^ Once heated, aerosols of e-liquids are even more complex mixtures, as some compounds can break down during the heating step in the device. For example, studies have shown that PG and G can break down into smaller aldehydes and carbonyls, such acrolein and formaldehyde,^9^ and sucralose can form harmful organochlorine compounds, and can result in higher PG and G degradation in e-liquids.^10^

Coolants are a class of molecules that produce a cooling sensation in the mouth and throat by activating the TRPM-8 receptor.^11^ Historically, the most important coolant has been menthol, but synthetic coolants like WS-23 (also *N*,2,3-trimethyl-2-propan-2-ylbutanamide, CAS No. 51115–67-4) and WS-3 (also N-ethyl-5-methyl-2-(propan-2-yl)cyclohexanecarboxamide, CAS No. 39711–79-0) have been shown to be present in e-cigarettes since at least 2019.^12^ Both WS-23 and WS-3 were developed by the Wilkinson-Sword company (which explains the WS naming convention) in the 1970–80s as menthol replacements in personal hygiene products, generating a similar cooling effect with lower volatility and no odor.^13^ One reason for the use of coolants is to facilitate inhalation and reduce the harshness of nicotine.^14^ This affects user behavior by allowing users to take longer and deeper puffs, increasing their nicotine uptake, and potentially leading to more frequent e-cigarette use.^15^ Recent studies on the contents of popular disposable devices, which accounted for > 60% of sales in the US market in 2025,^7^ demonstrate the almost ubiquitous presence of the synthetic coolant WS-23 (and the artificial sweetener neotame, see next paragraph), in many cases alongside the synthetic cooling agent WS-3.^3,5^ Few studies on the transfer rates of WS-23 and WS-3 to aerosol exist, but we recently reported carryover rates of 91–107% (n = 8) for WS-3 and 99% (n = 1) for WS-23 when using a refillable pod device.^3^ The synthetic coolant WS-3 was introduced in 2022 into so-called “non-menthol” cigarettes following statewide bans on menthol in Massachusetts and California,^16,17^ but updated California legislation resulted in the removal of WS-3-containing cigarettes from the state.^18^ A recent study determined 14–30% carryover of WS-3 from the WS-3-containing cigarettes to total particulate matter (TPM) collected on filter pads from machine smoking.^19^

Sweeteners are often added to nicotine and tobacco products to mask the bitterness of nicotine and enhance e-liquid flavors.^20^ In particular, artificial sweeteners have been detected in various tobacco products including cigarillos,^21^ SNUS and nicotine pouches.^22,23^ Most recently, we have reported on the prevalence of the artificial sweetener neotame – rated as 7,000–13,000 times sweeter than table sugar (sucrose)^24^ – in various e-cigarettes including those marketed as nicotine-free or nicotine analogue products.^5,25^ Neotame (also E961 or *N*-(*N*-(3,3-dimethylbutyl)-L-α-aspartyl)-L-phenylalanine 1-methyl ester) is derived from aspartame through N-alkylation,^24^ and one patent suggests neotame’s superior heat stability compared to sucralose.^26^ Beyond neotame, the use of the artificial sweetener sucralose – rated as 600 times sweeter than table sugar (sucrose)^24^ – has also been reported in e-cigarettes.^27^ Sucralose (also E955 or trichlorosucrose) is derived from sucrose with three hydroxy (-OH) groups substituted by chloro-substituents (-Cl), resulting in a sweet compound that cannot be metabolized in humans.^24^

Existing studies of neotame’s health effects focus on its impact on the digestive tract and have shown that neotame can cause death to intestinal epithelial cells, potentially compromising the integrity of the gut lining.^28^ In 2023, the World Health Organization’s (WHO) International Agency for Research on Cancer (IARC) classified aspartame, neotame’s structural analog, as possibly carcinogenic to humans, which was reaffirmed by the Food and Agriculture Organization (FAO) Joint Expert Committee on Food Additives (JECFA).^29,30^ Furthermore, studies have found that the presence of the sweeteners neotame and sucralose in e-liquid increases the release of heavy metals, resulting in higher concentrations in e-liquids and enhanced cytotoxicity in cell-based assays.^31^ While the main route of exposure for e-cigarette use is through inhalation rather than by ingestion, the latter is the more commonly studied route for artificial sweeteners due their common use in food products. As a result, there is a lack of knowledge on the effects of chronic inhalation of neotame and other sweeteners via e-cigarettes.

The effect of WS-23 on lung epithelial cells has been examined and shown to impair cytoskeletal function,^32^ adversely affect cell growth and morphology, and correlate with cytotoxicity in a concentration-dependent manner.^4^ Another in vitro study found that WS-23 and nicotine aerosols can induce goblet cell hyperplasia, likely impacting susceptibility to airway disease.^33^ However, there is a lack of knowledge on the effects of chronic inhalation of synthetic coolants such as WS-23 or WS-3.^34^

To support future studies that evaluate the risk of inhalational exposure to compounds commonly found in e-cigarettes and other tobacco products, it is important to determine the transfer rate of compounds from the e-liquid or tobacco product to the aerosol or smoke - what we refer to as the carryover - which is what users of these products are ultimately exposed to.^35^ While we have previously reported on the carryover of WS-23 and WS-3, these previous experiments were carried out in a refillable device and for a limited number of refill e-liquids.^3^

Therefore, this study aimed to quantify the carryover of the commonly found e-cigarette additives neotame and WS-23 directly from disposable devices to simulate user exposure. Ancillary analyses were carried out for menthol, nicotine, sucralose, and WS-3 carryover. To do so, popular disposable devices in the United States in 2025 were purchased and carryover experiments were carried out using an established method with an in-house built vaping machine and custom-made 3D-printed connectors.^36^

## MATERIALS AND METHODS

### Selection of Brands and Flavors

Popular e-cigarette brands and flavors were chosen based on the Discarded Pack Surveys commissioned by Altria. Three locations were surveyed (New York City;^37^ Washington, D.C.;^38^ California^39^) and the three most prevalent brands per location were selected: Air Bar, Breeze, Elf Bar (EB Create), Flum, Geek Bar, Lost Mary. Two flavors were selected per brand according to the most common flavors found in the sample and availability for purchase through online vendors. To account for potential effects of nicotine, two additional brands marketed to be either nicotine-free (Senza) or containing the nicotine analogue 6-methylnicotine (SpreeBar) were also purchased and tested. Samples were purchased between 11/2023–6/2026 from several online and local vendors (see Table 1).

### Vaping Carryover Experiments

Triplicate carryover experiments were conducted using an established method based on an in-house built vaping machine using cold-finger traps and a programmable puffing regime including number of puffs, interpuff-break, and flowrate.^36^ The setup consisted of a micro diaphragm pump (KNF Neuberger Inc., Trenton, NJ) attached to a needle valve, flow meter, two cold finger traps in series, and a custom 3D-printed connector to the e-cigarette device. The pump was connected to a DC power supply (Velleman, Fort Worth, TX) controlled by an Arduino Uno controller (Arduino, Somerville, MA). For each replicate, the device mass was measured before and after the experiment (Sartorius Practum, Göttingen, Germany) and the puffing regime was 20 puffs of 2.4 s at a flow rate of 1.85 L/min resulting in a puff volume of 74 mL, with a 30 s interpuff break. Cold finger traps were chilled with liquid nitrogen (Airgas, Radnor, PA). After 20 puffs were collected, the cold finger traps were removed from the liquid nitrogen and allowed to thaw. The traps were carefully rinsed with a total of 1 mL methanol (LC-MS grade, Fisher Scientific, Waltham, MA) and the solution was stored for analysis by gas and liquid chromatography. Carryover % was calculated per compound as $\frac{\backslash \text{raisebox}1ex\$\text{combined}\;\text{trap}\;\text{content}\;(\text{mg})\$/\backslash \text{raisebox}-1ex\$\text{mass}\;\text{difference}\;\text{of}\;\text{device}\;(\text{g})\$\;}{\text{compound}\;\text{content}\;\text{in}\;\text{neat}\;\text{e}-\text{liquid}\;(\text{mg}/\text{g})\;}\times 100$ (see Fig. 1).

### E-liquid content analysis

After triplicate vaping carryover experiments, the devices were disassembled using appropriate tools such as pliers and tweezers to collect e-liquid. All devices but Senza contained a sponge that held the liquid, which was isolated and compressed to expel the e-liquid into a vial for storage. For Senza, the device was carefully opened and the liquid directly collected using a Pasteur pipette.

### Gas Chromatography

Gas chromatography coupled with flame ionization detection (GC-FID) was used to quantify menthol, nicotine, WS-23, and WS-3, and to estimate PG/G ratio. Stored aerosol solutions were used as is, and approx. 40–50mg of extracted e-liquid from the devices was diluted with 1 mL methanol containing 1,4-dioxane at 1 g/L as internal standard. 1 μL of the methanolic solutions was injected into a Shimadzu GC-2010 (Columbia, MD) outfitted with an Agilent J&W DB-5 column (length 60 m, id 0.25 mm, 0.25 μm film; Agilent, Santa Clara, CA) and using helium as the carrier gas (Airgas, Radnor, PA). The injector temperature was 250°C and the detector temperature was 325°C. The oven temperature program was: hold at 30°C for 7 min; ramp to 50°C at a rate of 10°C/min; hold for 20 min; ramp to 310°C at a rate of 10°C/min; hold for 12 min. Calibration curves for menthol (≥ 99% purity), WS-23 (> 98%), WS-3 (> 98% ;all TCI America, Portland, OR), L-nicotine (99+%; Thermo Scientific, Waltham, MA), and racemic 6-methylnicotine (97%, Santa Cruz Biotechnology, Dallas, TX) with at least 5 points were constructed using commercial standards (see Table S4 for LOD, LOQ, and %RSD). PG/G ratio was estimated solely based on peak area ratio and rounded to the closest multiple of 5.

### Liquid Chromatography

Liquid chromatography coupled with tandem mass spectroscopy (LC-MS/MS) was used to test for the presence of 12 sweeteners using an established method, and to quantify the detected neotame and sucralose.^5^ Both the stored aerosol solutions and prepared solutions for GC-FID analysis were diluted 100-fold using methanol. 10 μL of the methanolic solutions were injected into a Thermo Vanquish LC coupled to a Quantis MS (both Thermo Scientific, Waltham, MA) outfitted with an Accucore RP-MS column (50 mm × 2.1 mm id, 2.6 μm particle size, Thermo Scientific) at an oven temperature of 40°C. The mobile phase was 0.1% formic acid in water (phase A) and 0.1% formic acid in acetonitrile (phase B; both Optima grade, Fisher Scientific). The flow rate was 0.4 ml/min and the mobile phase gradient was as follows: Hold at 95% A for 0.5 min, ramp to 5% A in 2 min, hold at 5% A for 2 min, ramp to 95% A in 0.1 min, hold at 95%A for 3.9min; total run time: 8 min. For MS/MS, heated electrospray ionization (H-ESI) was used with 3,500 V in positive mode and 2,500 V in negative mode. Selected reaction monitoring was carried out based on direct injections of the 12 sweeteners and polarity and m/z values for precursor and quantifying and confirming products reported previously.^5^ Calibration curves for neotame (≥ 98%, Sigma Aldrich, Burlington, MA) and sucralose (> 98%, TCI) with at least 5 points were constructed using commercial standards (see Table S4 for LOD, LOQ, and %RSD).

### Statistical analysis

Pearson linear regression, Spearman nonparametric correlation,^40^ and one-way ANOVA with Bonferroni multiple comparisons were carried out using GraphPad Prism V11.1.0 (224; Dotmatics, Boston, MA).

## RESULTS

Neotame was detected in the e-liquids contained in all 17/17 tested devices at a range of 0.05–2.73 mg/g and a mean (SD) concentration of 0.99 mg/g (0.81; see Table 1). Carryover experiments showed a range of neotame carryover of 15%−64% and a mean (SD) carryover of 40% (13%; see Table 2).

WS-23 was detected in the e-liquids contained in all 17/17 tested devices at a range of 3.52–35.0 mg/g, and a mean (SD) concentration of 19.7 mg/g (8.49). Carryover ranged from 71%−97%, with a mean (SD) carryover of 84% (9%).

Menthol was present in 9/17 devices tested at a range of 0.31–10.5 mg/g and a mean (SD) concentration of 4.2 mg/g (4.48) in the e-liquid. Carryover ranged from 69%−91%, with a mean (SD) carryover of 81% (8%).

The 11/17 devices labeled to contain nicotine did contain nicotine at a range of 23.1–54.2 mg/g and a mean (SD) concentration of 31.0 mg/g (10.8). Carryover ranged from 64%−101%, with a mean (SD) carryover of 88% (12%).

Sucralose was detected in 2/17 devices at 6.75 mg/g and 11.8 mg/g. Measured carryover percentages were 26% and 54%, resulting in a mean (stdev) carryover of 40% (21%).

WS-3 was detected in 4/17 devices tested at a range of 4.42–7.67 mg/g and a mean (stdev) concentration of 6.4 mg/g (1.56). Carryover ranged from 81%−103%, with a mean (stdev) carryover of 88% (10%).

Statistical analysis was carried out to identify potential correlations between individual compound content and its carryover-%, and PG content of the respective e-liquid and compound carryover-%. Neither Pearson linear regression nor Spearman nonparametric correlation demonstrated a correlation between compound concentration, or PG content of the liquid, and the respective compound carryover (Tables S1 & S2). One exception was for nicotine, where the analysis of the full dataset of nicotine-containing e-liquids (n = 11) indicated a significant negative relationship between nicotine content and nicotine carryover-% (Pearson R^2^= 0.65 with p = 0.003; Spearman ρ=−0.66 with p = 0.032; Table S1, Figure S4). However, this relationship was driven by two data points > 50 mg/g and a re-analysis excluding these 2 points (n = 9) resulted in non-significance (Pearson R^2^= 0.059 with p = 0.53; Spearman ρ=−0.37 with p = 0.32; Table S1). One way ANOVA with Bonferroni multiple comparisons showed no significant differences for neotame or WS-23 carryover between devices containing nicotine, no nicotine, or 6-methylnicotine (Table S3).

## DISCUSSION

### Neotame and sucralose

Based on our results, there is substantial carryover of neotame from e-liquids contained in disposable devices into the aerosol generated from them with an average of 40% of neotame reaching users. These results are somewhat aligned with a 2023 industry presentation on neotame carryover, where authors found a 54.66% carryover from a “disposable device” - it remains somewhat unclear what type of device was used in that study.^41^ However, our data also suggests a rather wide range of carryover effectiveness between devices, ranging from 15–64% (see Table 2). While some devices seemed to produce a somewhat consistent carryover of neotame between flavors, others varied between different flavors (see Table 2). The presence or absence of nicotine or the nicotine analogue 6-methylnicotine did not impact neotame carryover. Statistical analysis further revealed no correlation between the absolute amount of neotame in the e-liquid and carryover-%, as well as no correlation between PG content and carryover.

The observed carryover rates of neotame ranging from 15–64% raise several questions: (1) Is the aerosolization process simply not effective enough and the remaining neotame ultimately concentrates within the e-liquid over time? (2) Does some of the neotame condense or crystallize on the path of the aerosol from the coil to the exit of the device as we have shown previously for sucralose in earlier-generation devices?^36^ (3) Or is the low carryover rate a result of thermal (or other) breakdown of the neotame molecules during aerosolization, which would also lead to lower observed carryover.

We did not further pursue (1) and (2) due to material (device) constraints. Regarding (3), we did not further test the collected aerosol for potential neotame breakdown products; however, we did not observe additional, unexplained peak by GC-MS, suggesting that if thermal breakdown were to happen it would not yield compounds detectable by GC-MS. One approach to better understand potential thermal breakdown products of neotame is to consider the thermal breakdown products of its structural analogue aspartame, which, as outlined, only differs from neotame by the presence of a 3,3-dimethylbutyl group, with the rest of the molecules being otherwise identical (compare Figs. 2 & 3).^5^ Thermal degradation of aspartame yields a mixture of compounds including the ring-shaped compound diketopiperazine (DKP), methanol, and others (see Figure 2),^42,43^ yet one animal study found no adverse effects from oral administration of DKP over 104 weeks.^44^

A 2003 Chemical and Technical Assessment by the Joint FAO/WHO Expert Committee on Food Additives (JECFA) assessed neotame and its potential degradation products, based on some of the known breakdown products of aspartame (compare Figures 2 & 3). However, that report focused on neotame use in foods such as beverages and milk products, including at various pH and temperature conditions up to 87.8 °C.^45^ Because the chemical environment in beverages and milk products is starkly different from e-liquids, other breakdown products of neotame can reasonably be expected during aerosolization given the different environment and higher temperatures in e-cigarettes. Taken together, the inhalational safety of neotame and its thermal breakdown products remains unclear and warrants further investigation given its prevalence in popular e-cigarettes.

Interestingly, while Lost Mary products contained the lowest neotame concentrations in the tested sample (0.07–0.16 mg/g), we detected sucralose as an additional sweetener at much higher concentrations (6.75–11.8 mg/g). To our knowledge, this is the first report describing the co-use of these two sweeteners in e-cigarette products. While combining several artificial sweeteners in food products is a common practice to better replicate the sensory perception of the replaced sucrose,^46^ it seems that this practice is now also being employed in e-cigarettes. Mixing two or more sweeteners further increases the complexity of e-cigarette products and raises additional questions about the inhalational safety of the products. For example, heating sucralose in e-cigarettes has been reported to lead to the formation of harmful organochlorine compounds and to result in higher PG and G degradation in e-liquids.^10,47^ Further questions remain about how addictive “sweetness-optimized” e-cigarettes are and what other kinds of chemical or physiological interactions could occur, an area that is still poorly understood in e-liquids and tobacco products in general.^48^

### Synthetic coolants, menthol, and nicotine

Mean carryover rates for the two synthetic coolants WS-23 (84%) and WS-3 (88%), menthol (81%), and nicotine (88%) were substantially higher than for the artificial sweeteners (Table 2). These results align with prior reports of high carryover (91–100%) of these four compounds in a refillable pod system and Juul using the same setup, albeit at slightly lower transfer effectiveness,^3,49^ and a study using a different setup that found menthol carryover of 86–107%.^50^ The obvious difference between the carryover of the artificial sweeteners of ~40% and values >80% for WS-23, WS-3, menthol, and nicotine raises the question of what influences the carryover effectiveness of different compounds present in e-liquids. Two studies have outlined parameters that influence carryover effectiveness in electronic cigarettes including (1) physico-chemical properties of the molecules in question such as boiling point and vapor pressure, (2) device characteristics such as coil temperature and e-liquid makeup, (3) the puffing regime used including puff duration and volume,^51^ but we propose to also include (4) the reactivity of the compounds in question during heating, or with other additives or the device (coil, wick, sponge) which could lead to compound breakdown and a lower observed carryover.

Two physico-chemical properties used to define volatile organic compounds (VOCs) – usually in regulatory contexts – are vapor pressure and boiling point. For example, regulatory limits for VOCs set by the US EPA, the European Union, and Health Canada include a vapor pressure <0.1 mm Hg (<0.013 kPa)^52^ or <0.01 kPa^53^ (both at 20 °C); or a boiling point of >216 °C^54^ or >250 °C.^55^ The four compounds with observed carryover of >80% are somewhat close to said regulatory limit at least in terms of boiling point (vapor pressure 7.2e-6 – 5.3e-3 kPa; bp. 212–341 °C; see Table 3) and thus have a certain degree of volatility. While there is limited knowledge about the temperatures within disposable e-cigarettes, cutoff points to prevent overheating, and differences among brands and device settings, the cutoff point for the Juul system has been reported as approx. 215 °C.^56^ Assuming that disposable devices operate in a temperature regime that is somewhat comparable, the boiling points of the 4 compounds are somewhat near their boiling points during heating.

On the other hand, the boiling points of the two artificial sweeteners neotame and sucralose are both >500 °C and vapor pressures are 8–12 orders of magnitude lower than WS-23, WS-3, menthol, and nicotine. This could serve as one explanation for the observed lower carryover of neotame and sucralose. In addition, the Henry constants follow a similar trend. The Henry constant describes the tendency of compounds to move from a liquid phase to the gas phase, with smaller numbers suggesting more of the compound remains in the liquid phase; it should also be noted that the data presented in Table 3 is for water and not a PG/G system.

Regarding device characteristics, while we did not test for device characteristics such as coil resistance or wattage, most devices used 3.7V Li-ion batteries, but this number alone is not sufficient to determine device power output; and regarding the puffing regime, we used a similar puffing regime for all experiments.

Regarding compound stability or reactivity during heating, one Chinese study out of a tobacco industry laboratory examined the thermal stability of pure WS-23 and WS-3 by pyrolysis-gas chromatography (Py-GC/MS) and thermogravimetry.^58^ In the thermogravimetric studies, authors found an onset of weight loss at 98.7°C and maximum weight loss at 206.5°C for WS-23, and for WS-3, an onset of weight loss at 145.2°C and maximum weight loss at 206.5°C. The presented Py-GC/MS data suggests specific breaking points in the molecules (C-C bond adjacent to the carbonyl group of WS-23; and the amide bond in WS-3) as well as alkyl, carbonyl, and amino compounds for both WS-23 and WS-3, and ether compounds for WS-3. These are first indications of the relative instability of WS-23 and WS-3 when heated, although the data we present here suggests that most of the synthetic cooling agents in this study did indeed reach the aerosol - and thus did not break down. Nonetheless, the ubiquitous use of WS-23 in e-liquids should raise concerns, especially when using high-powered devices. Gaining a better understanding of potential breakdown products of WS-23 and other popular additives is important to better assess chronic inhalation effects.

### Limitations

Several limitations exist: Here, we only determined the e-liquid content contained in the disposable devices after taking 3 × 20 puffs from the device. While most devices indicate the availability of several thousand puffs contained in them, using the device for 60 puffs before testing its content might have resulted in under- or overestimation of the actual e-liquid concentrations of the 6 compounds, and consequently, the carryover percentages. However, the reported carryover results are generally aligned with prior studies, and few carryover results were >100%, suggesting that compound e-liquid concentrations were at least not underestimated. Alternatively, testing devices before use and then carrying out the carryover experiments with a second device of the same brand and flavor could have potentially been more problematic given uncertainty around device-to-device deviati ons. Ultimately, this limitation does not affect the key finding of the study that users of the tested disposable devices are exposed to both the artificial sweetener neotame and the synthetic coolant WS-23, independent of flavor variety and whether the devices contained nicotine, 6-methylnicotine, or no nicotine at all.

Another study limitation is that a standardized puffing regime as used here will never be able to simulate actual user behavior such as puff length, puff volume, or proportion that is inhaled into the lungs (vs. what remains in the oral cavity). As a result, the values determined here must be considered as one data point on a broader spectrum of exposure to these compounds.

Lastly, we observed differences in carryover for some compounds in products of the same brand, while carryover for other compounds was similar (see Table 2, e.g., Breeze: nicotine; Geek Bar: Neotame; Lost Mary: several compounds). It is unclear what the reasons for these differences are. We speculate that one reason could be the “disposable” nature of these devices, which could result in the use of low-quality materials inside the device – both to store and to aerosolize the liquid – to limit cost.

### Outlook

This work demonstrates the continued proliferation of the potent artificial sweetener neotame and the synthetic coolant WS-23 in popular disposable e-cigarettes on the US market. Importantly, it establishes that a significant amount of these compounds also reaches users of these devices – on average 40% of the neotame and >80% of the WS-23 contained in the devices. These findings raise several important questions: What are the health implications of chronic inhalational exposure to these compounds? How might these compounds break down in the variety of available e-cigarettes (variable temperature, variable air flow, etc.) and what are the potential health effects of chronic exposure to these breakdown products? How these compounds interact with nicotine and how might they modulate the addictiveness of the tested devices?

## CONCLUSION

The goal of this work is to support and inform future studies that will be required to better understand the implications of the changing landscape of the US disposable e-cigarette market, within which neotame and WS-23 now seem ubiquitous ingredients. This work begins to establish a foundation for future toxicological and pharmacological studies informing human health risk by reporting on the carryover of 6 compounds from e-liquid contained in disposable devices to aerosol thereof, including for neotame and WS-23. We also report finding at least one brand in which 2 sweeteners (neotame, sucralose) are present, potentially to “optimize” the sweet effect while suppressing any negative effects such as bitterness. Future work is needed to better understand the stability of these additives during storage and heating of the liquids, to better understand the health effects of chronic inhalation of the compounds and their potential breakdown products, and to understand how their presence affects the abuse liability of these products.

## Supplementary Material

Supplementary Files

This is a list of supplementary files associated with this preprint. Click to download.

2026110NeotameCarryoverSIramhceixmSOEMB.docx

## Figures and Tables

**Figure 1:**
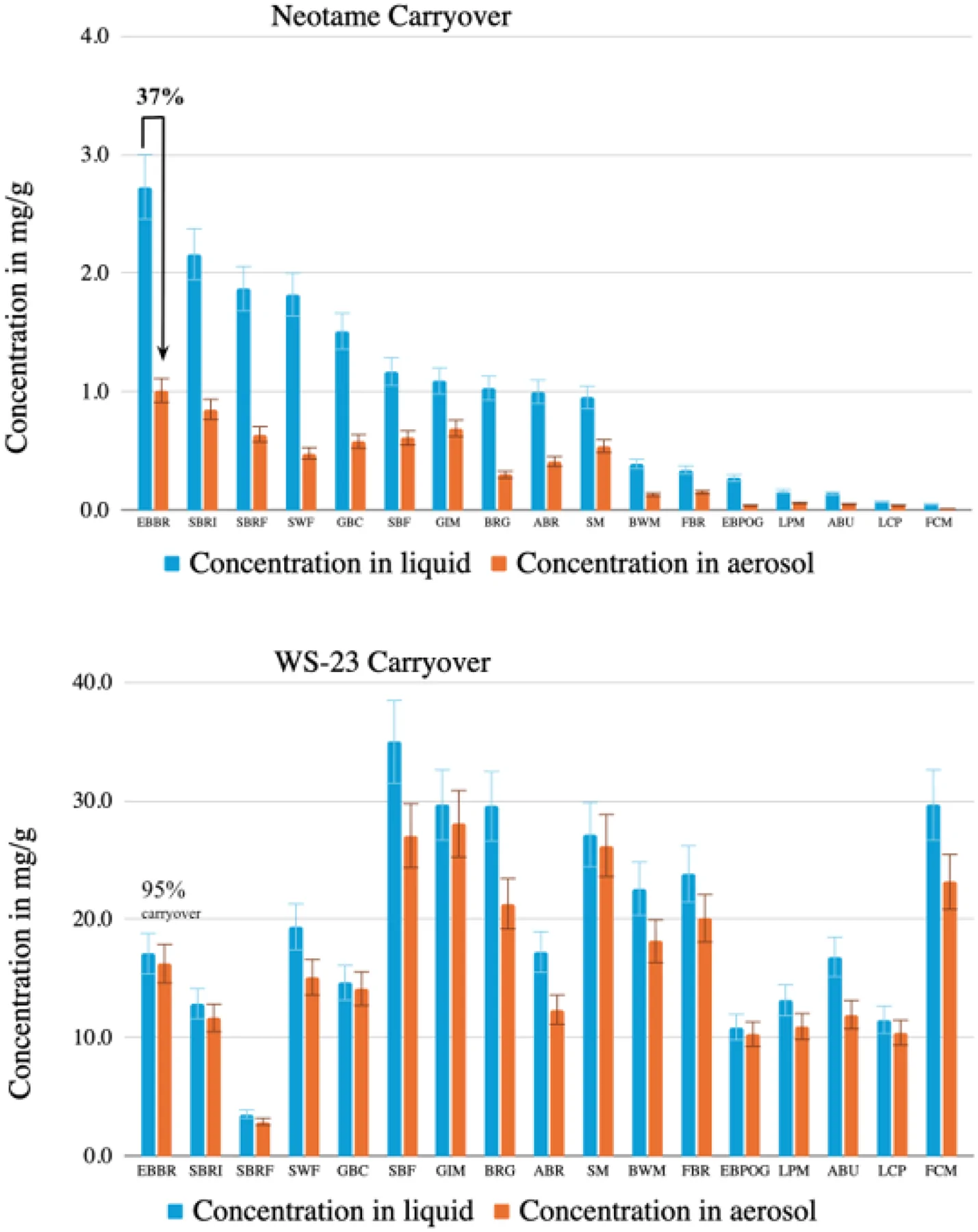
Concentrations of neotame and WS-23 in neat e-liquid (blue) and trapped aerosol (red), with carryover-% indicated for first data pair. Error bars represent standard deviation of the mean, all N = 3. Abbreviations, left to right: EBBR - Elf Bar Blue Razz; SBRI - Spree Bar Blue Razz Ice; SBRF - SpreeBar Rainbow Fruit; SWF - Sensa Watermelon Frost; GBC - Geek Bar Cherry; SBF - Sensa Blueberry Frost; GIM - Geek Bar Icy Mint; BRG - Breeze Grape; ABR - Air Bar Blue Razz Icy; SM - Sensa Mint; BWM - Breeze Watermelon Mint; FBR - Flum/Float Blue Razz Icy; EBPOG - Elf Bar Passion Orange Guava; LPM - Lost Mary Pineapple Mango; ABU - Air Bar Unicorn; LCP - Lost Mary Cherry Peach Lemonade; FCM - Flum/Float Cool Mint.

**Figure 2:**
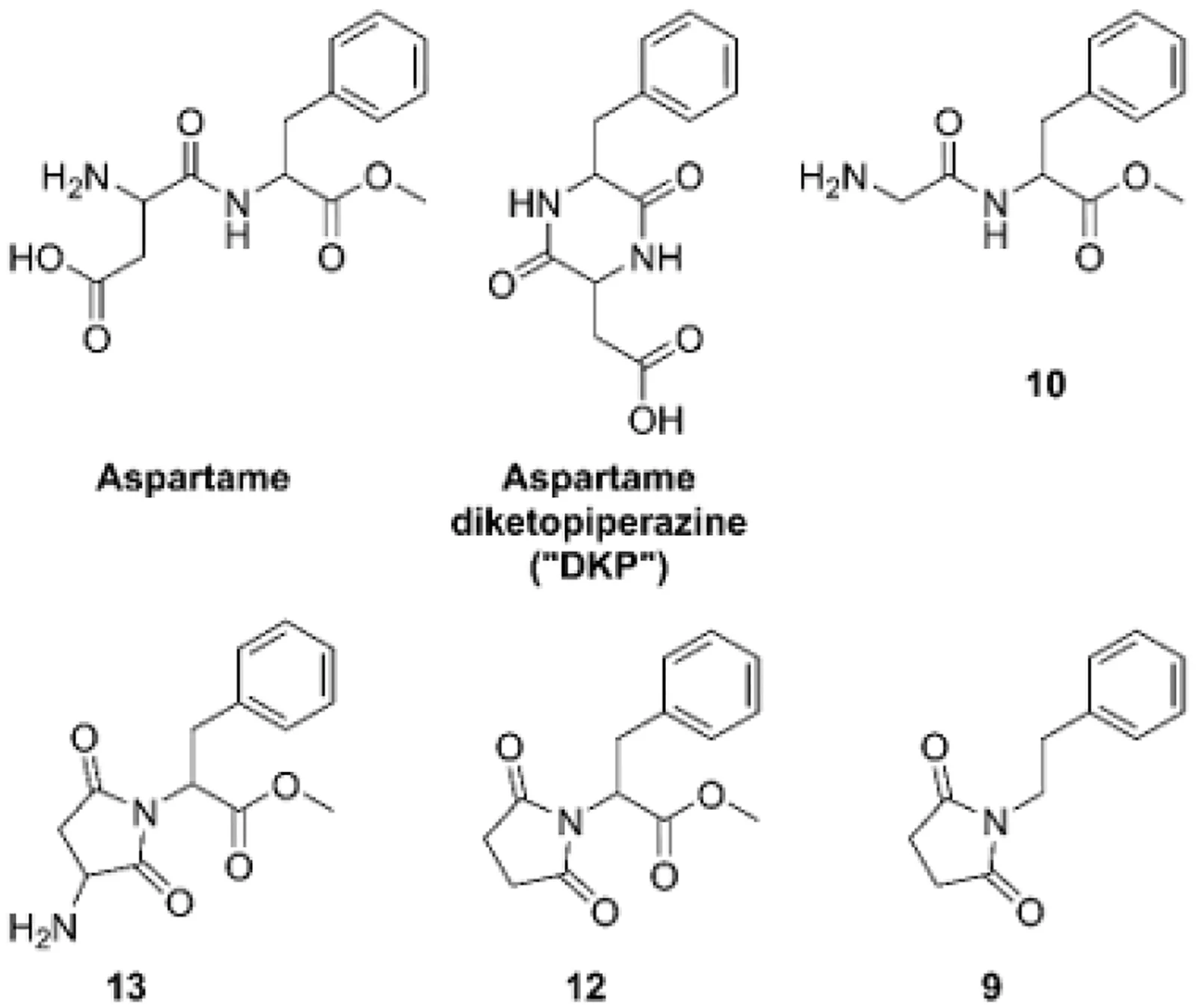
Structure of aspartame, DKP, and several high-molecular weight pyrolysis products, adapted from Galetti *et al*. (1995).^42^ Abbreviations: DKP: diketopiperazine; 10: 2-(2-Aminopropionylamino)-3-phenylpropionic acid methyl ester; 13: 2-(3-Amino-2,5-dioxopyrrolidin-1-yl)-4-phenylbutyric acid methyl ester; 12: 2-(2,5-Dioxopyrrolidin-I-yl)-4-phenylbutyric acid methyl ester; 9: I-Phenetylpyrrolidine-2,5-dione.

**Figure 3:**
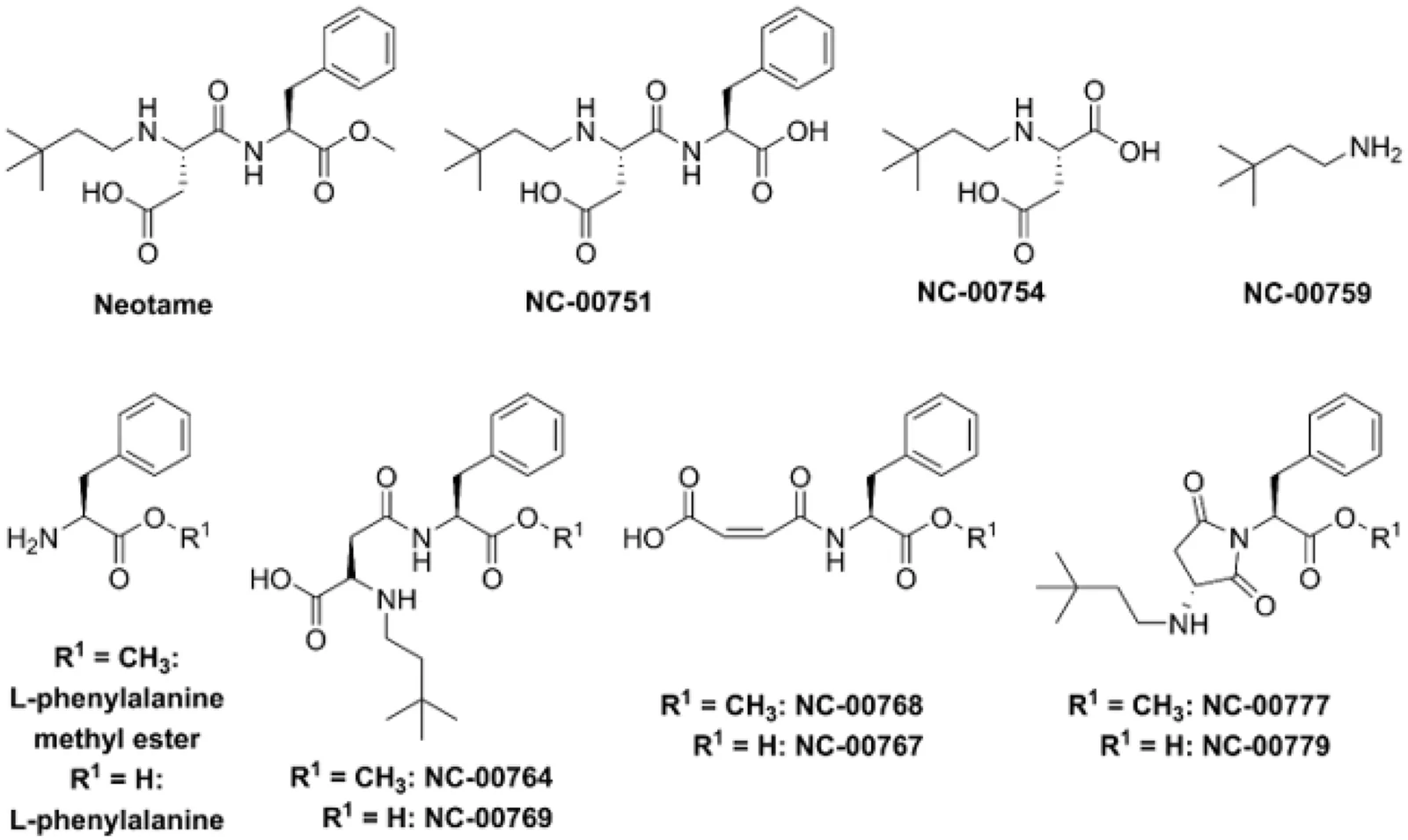
Structure of neotame and a range of potential degradation products during thermal processing for beverage and dairy products based on identified aspartame degradation products; adapted from a 2003 Chemical and Technical Assessment by the Joint FAO/WHO Expert Committee on Food Additives (JECFA).^45^ Abbreviations: NC-00751: N-[N-(3,3-dimethylbutyl)-L-α-aspartyl]-L-phenylalanine; NC-00754: N-(3,3-dimethylbutyl)-L-aspartic acid; NC-00759: 3,3-dimethylbutylamine; NC-00769: N-[N-(3,3-dimethylbutyl)-L-β-aspartyl]-L-phenylalanine; NC00764: NC-00769 1-methyl ester; NC-00767: N-Fumarylphenylalanine; NC-00768: NC-00767 1-methyl ester; NC-00779: N-[N-(3,3-dimethylbutyl)-L-aspartamidyl]-L-phenylalanine; NC-00777: NC-00779 1-methyl ester.

**Table 1:** Maker and labelled flavor of tested disposable devices, estimated propylene glycol to glycerol (PG/G) ratio^a^, and measured concentrations of neotame, WS-23, menthol, nicotine, sucralose, and WS-3 in tested e-liquids reported as mean (SD) in mg/g, and purchase date and vendor^d^.

Maker	Flavor	PG/G ratio	Neotame	WS-23	Menthol	Nicotine	Sucralose	WS-3	Purchase Date and vendor^d^
AirBar Nex	Blue Razz Icy	65/35	1.00 (0.21)	17.2 (0.08)	0.31 (0.01)	29.5 (0.17)			9/2024 EBD
AirBar Nex	Unicorn	70/30	0.14 (0.01)	16.8 (0.04)		24.4 (0.08)			
Breeze	Grape	55/45	1.03 (0.04)	29.6 (0.21)		50.3 (0.39)			9/2024 EJDB
Breeze	Watermelon Mint	55/45	0.39 (0.01)	22.6 (0.22)		54.2 (0.58)			
ElfBar	Passionfruit Orange Guava	55/45	0.27 (0.01)	10.9 (0.08)		28.4 (0.20)			9/2024 EBD
ElfBar Create	Blue Razz Ice	65/35	2.73 (0.26)	17.1 (0.76)	0.49 (0.02)				
Flum	Blue Razz Icy	55/45	0.34 (0.02)	23.8 (0.56)	0.49 (0.06)	27.2 (0.63)			6/2026 VD
Flum	Cool Mint	65/35	0.05 (0.01)	29.7 (0.21)	9.84 (0.07)	30.4 (0.17)		4.42 (0.03)	11/2023 MIP
Geek Bar	Black Cherry	60/40	1.51 (0.05)	14.6 (0.21)	0.61 (0.01)	23.1 (0.35)			6/2026 VD
Geek Bar	Icey Mintz	60/40	1.09 (0.01)	29.7 (0.08)	6.23 (0.01)	23.8 (0.04)			
Lost Mary	Cherry Peach Lemonade	65/35	0.07 (0.00)	11.5 (0.03)		24.3 (0.05)	11.8 (0.43)		6/2026 LMD
Lost Mary	Pineapple Mango	60/40	0.16 (0.00)	13.2 (0.02)		25.4 (0.09)	6.75 (0.37)		
Sensa	Blueberry Frost	65/35	1.17 (0.13)	35.0 (0.05)	8.63 (0.01)			7.67 (0.02)	7/2024 NCCS
Sensa	Mint	60/40	0.95 (0.03)	27.2 (0.29)	10.5 (0.09)			6.02 (0.05)	
Sensa	Watermelon Frost	70/30	1.82 (0.23)	19.4 (0.58)	1.06 (0.05)			7.66 (0.05)	
SpreeBar	Blue Razz Ice	60/40	2.16 (0.45)	12.9 (0.25)		[3.62 (0.08)]^c^			1/2024 WSV
SpreeBar	Rainbow Fruit	60/40	1.87 (0.04)	3.5 (0.02)		[3.91 (0.01)]^c^			
	**MEAN**		*0.99*	*19.67*	*4.24*	*31.0* ^d^	*9.27*	*6.44*	
	**SD**		*0.81*	*8.49*	*4.48*	*10.8* ^d^	*3.56*	*1.56*	
	**Minimum**		*0.05*	*3.52*	*0.31*	*23.1* ^d^	*6.75*	*4.42*	
	**Maximum**		*2.73*	*35.0*	*10.5*	*54.2* ^d^	*11.8*	*7.67*	

a estimation based on integration of PG and G peaks on FID chromatogram and rounded to nearest multiple of 5;

b Abbreviations: EBD: ebdesigndisposablevape.com, EJDB: ejuicedb.com, LMD: lostmarydirect.com, MIP: mipod.com, NCCS: local North Carolina convenience store, VD: Vapordna.com, WSV: westsidevapor.com;

c square brackets denotes 6-methylnicotine content;

d Results compiled only for nicotine (n = 11).

**Table 2:** Quantified carryover-% of neotame, WS-23, menthol, nicotine, sucralose, and WS-3 from liquid to aerosol.

Maker	Flavor	Neotame	WS-23	Menthol	Nicotine	Sucralose	WS-3
AirBar Nex	Blue Razz Icy	41%	72%	74%	87%		
AirBar Nex	Unicorn	40%	71%		86%		
Breeze	Grape	29%	72%		84%		
Breeze	Watermelon Mint	34%	80%		64%		
ElfBar	Passionfruit Orange Guava	15%	94%		101%		
ElfBar Create	Blue Razz Ice	37%	95%	91%			
Flum	Blue Razz Icy	45%	84%	77%	91%		
Flum	Cool Mint	29%	78%	72%	96%		86%
Geek Bar	Black Cherry	38%	97%	85%	101%		
Geek Bar	Icey Mintz	64%	95%	86%	98%		
Lost Mary	Cherry Peach Lemonade	60%	91%		101%	54%	
Lost Mary	Pineapple Mango	36%	83%		92%	26%	
Senza	Blueberry Frost	52%	77%	69%			81%
Senza	Mint	57%	97%	87%			103%
Senza	Watermelon Frost	26%	78%	83%			81%
SpreeBar	Blue Razz Ice	39%	91%		72%		
SpreeBar	Rainbow Fruit	34%	82%		77%		
	**MEAN**	*40%*	*84%*	*81%*	*88%*	*40%*	*88%*
	**SD**	*13%*	*9%*	*8%*	*12%*	*21%*	*10%*
	**Minimum**	*15%*	*71%*	*69%*	*64%*	*26%*	*81%*
	**Maximum**	*64%*	*97%*	*91%*	*101%*	*54%*	*103%*

**Table 3:** CAS number, melting point, boiling point, vapor pressure, and Henry’s constant for compounds quantified in aerosol of disposable e-cigarettes. Experimental values except where indicated. Data source: U.S. EPA Estimation Programs Interface Suite^™^ V.1.1.0.^57^

	Artificial sweeteners		Synthetic coolants			
	neotame	sucralose	WS-23	WS-3	menthol	nicotine
**CAS number**	165450-17-9	56038-13-2	51115-67-4	39711-79-0	89-78-1	54-11-5
**Melting point** (°C)	317 (estimated)	130	60	88	43	−79
**Boiling point** (°C)	572 (estimated)	564 (estimated)	278 (estimated)	341	212	247
**Vapor pressure** (mm Hg) (kPa)	2.7e-11 3.6e-12	1.3e-14 1.7e-15 (estimated)	2.9e-3 3.8e-4 (estimated)	5.4e-5 7.2e-6	0.06 8.0e-3	0.04 5.3e-3
**Henry constant** (Atm-m^3^/mol) *(all estimated)*	2.3e-17	4.0e-19	1.8e-7	1.8e-7	2.6e-5	3.0e-9
